# Syntheses and crystal structures of the quaternary thio­germanates Cu_4_FeGe_2_S_7_ and Cu_4_CoGe_2_S_7_

**DOI:** 10.1107/S2056989020007872

**Published:** 2020-06-19

**Authors:** Andrew J. Craig, Stanislav S. Stoyko, Allyson Bonnoni, Jennifer A. Aitken

**Affiliations:** a Department of Chemistry and Biochemistry, Duquesne University, 600 Forbes Ave, Pittsburgh PA 15282, USA

**Keywords:** crystal structure, diamond-like, thio­germanate, quaternary sulfide

## Abstract

The quaternary thio­germanates Cu_4_FeGe_2_S_7_ (tetra­copper iron digermanium hepta­sulfide) and Cu_4_CoGe_2_S_7_ (tetra­copper cobalt digermanium hepta­sulfide) were prepared in evacuated fused-silica ampoules *via* high-temperature, solid-state synthesis using stoichiometric amounts of the elements at 1273 K. These isostructural compounds crystallize in the Cu_4_NiSi_2_S_7_ structure type, which can be considered as a superstructure of cubic diamond or sphalerite. The monovalent (Cu^+^), divalent (Fe^2+^ or Co^2+^) and tetra­valent (Ge^4+^) cations adopt tetra­hedral geometries, each being surrounded by four S^2−^ anions. The divalent cation and one of the sulfide ions lie on crystallographic twofold axes. These tetra­hedra share corners to create a three-dimensional framework structure. All of the tetra­hedra align along the same crystallographic direction, rendering the structure non-centrosymmetric and polar (space group *C*2). Analysis of X-ray powder diffraction data revealed that the structures are the major phase of the reaction products. Thermal analysis indicated relatively high melting temperatures, near 1273 K.

## Abstract 2

The isostructural crystal structures of Cu_4_FeGe_2_S_7_ and Cu_4_CoGe_2_S_7_ were solved and refined. All the metal cations form *M*S_4_ tetra­hedra and share corners to create a three-dimensional, non-centrosymmetric structure.

## Chemical context   

The title compounds belong to the family of quaternary thio­germanates, which can be constructed from different [Ge_x_S_y_]^z-^ building blocks, such as [GeS_4_]^4−^ (Aitken *et al.*, 2001 ▸) and [Ge_2_S_6_]^4−^ (Choudhury *et al.*, 2015 ▸). Two GeS_4_ tetra­hedra can share a corner to create [Ge_2_S_7_]^6−^ units, which are featured in the title compounds. Cu_4_FeGe_2_S_7_ and Cu_4_CoGe_2_S_7_ also belong to the family of diamond-like semiconductors (DLSs), with structures that can be derived from the cubic or hexa­gonal (Frondel & Marvin, 1967 ▸) forms of diamond. The synthesis of new diamond-like materials is guided by valence electron principles and Pauling’s rules, and the resulting DLSs can be binary, ternary, or quaternary, depending on the number of elements employed in the reaction (Parthé, 1964 ▸; Pamplin, 1981 ▸; Goryunova, 1965 ▸). Increasing the number of elements in the formula allows for greater tunability of the material’s properties; thus, quaternary DLSs are a particularly appealing class of materials. As a result of their technologic­ally relevant properties, these materials are of inter­est for a number of applications, such as solar cells (Ito & Nakazawa, 1988 ▸; Heppke *et al.*, 2020 ▸; Liu *et al.*, 2018 ▸), batteries (Brant, Devlin *et al.*, 2015 ▸; Kaib *et al.*, 2013 ▸) and magnetic devices (Wintenberger, 1979 ▸; Greenwood & Whitfield, 1968 ▸). Furthermore, owing to their inherently non-centrosymmetric structures, DLSs are attractive candidates for infrared non-linear optical (IR–NLO) devices that make use of second-harmonic generation (SHG) crystals (Ohmer & Pandey 1998 ▸): only crystals that lack an inversion center can exhibit SHG.

IR–NLO materials are used to shift the radiation of lasers to more suitable wavelengths for use in military (Hopkins 1998 ▸), medical (Stoeppler *et al.*, 2012 ▸) and industrial applications (Bamford *et al.*, 2007 ▸). Currently, ternary DLSs, most of which are sulfides, dominate the market of SHG crystals for use in the infrared (Ohmer & Pandey, 1998 ▸). Yet the current commercially available IR–NLO materials suffer from serious drawbacks, such as low laser-induced damage thresholds (LIDTs) and multi-photon absorption (Schunemann, 2007 ▸). Turning attention to the discovery of new quaternary DLSs provides a reliable route to next-generation IR–NLO materials that allows for greater control of the material’s properties. Compounds such as Li_2_CdGeS_4_ (Brant, Clark *et al.*, 2014 ▸), Li_2_MnGeS_4_ (Brant, Clark *et al.*, 2015 ▸), and Li_4_HgGe_2_S_7_ (Wu, Yang & Pan, 2017 ▸) have shown potential to outperform currently used ternary IR–NLO crystals. These DLSs have shown promising SHG capabilities, as well as resilience to high powered lasers, a necessity to broaden future usage (Hopkins, 1998 ▸). For these reasons, we were motivated to investigate the Cu–Fe–Ge–S and Cu–Co–Ge–S systems for new DLSs.

## Structural commentary   

**Figure 1:**
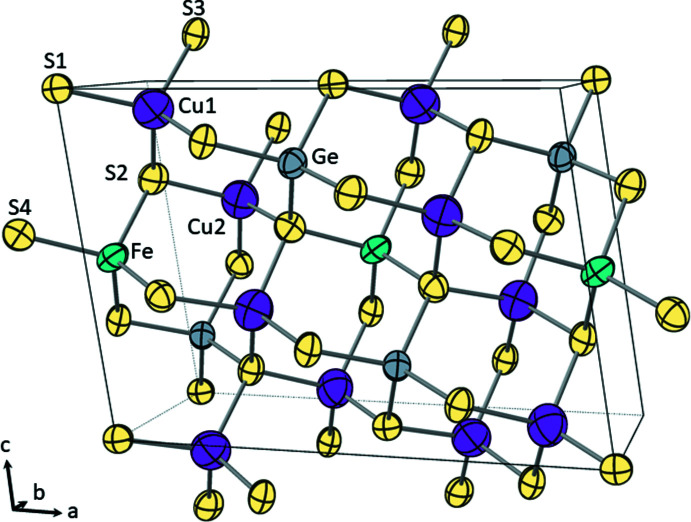
The structure of Cu_4_FeGe_2_S_7_ with crystallographically unique ions labeled. Ellipsoids are shown at 99% probability. Cu_4_CoGe_2_S_7_ is isostructural and has similar atomic displacement parameters.

**Figure 2:**
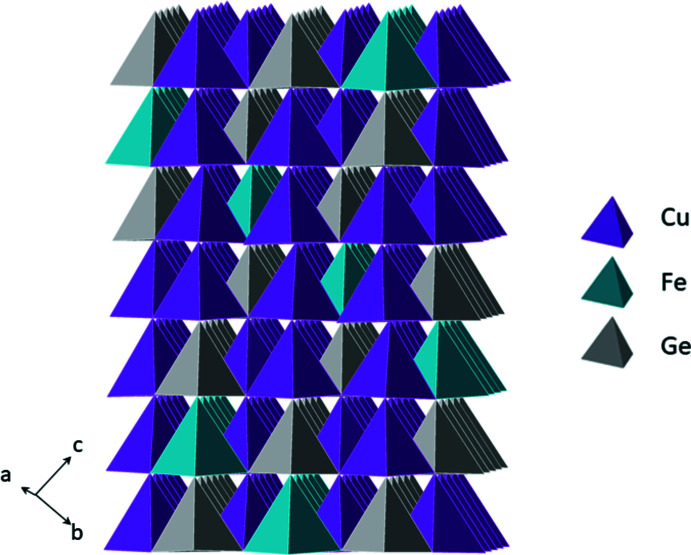
Polyhedral view of Cu_4_FeGe_2_S_7_ showing the polar nature of the structure.

**Figure 3:**
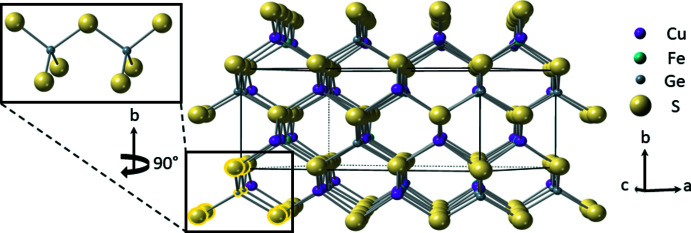
The ‘honeycomb’ pattern found in Cu_4_FeGe_2_S_7_, a characteristic of DLSs. Highlighted in the bottom left corner is one of the [Ge_2_S_7_]^6−^ subunits that forms in this structure.

The title compounds, Cu_4_FeGe_2_S_7_ (I) and Cu_4_CoGe_2_S_7_ (II), are isostructural and crystallize in the non-centrosymmetric, monoclinic space group *C*2 (No. 5) with the Cu_4_NiSi_2_S_7_ structure type (Schäfer *et al.*, 1980 ▸). The structure contains two crystallographically unique Cu^+^ ions, one divalent metal (Fe or Co) sited on a crystallographic twofold axis, one Ge^4+^ cation and four S^2−^ anions (one with site symmetry 2) (Fig. 1 ▸). The sulfide anions create a ‘cubic’ close-packed array and the cations reside in one-half of the tetra­hedral holes; these tetra­hedra share corners to form a three-dimensional network. Two GeS_4_ tetra­hedra share corners to form (Ge_2_S_7_)^6−^ subunits that are isolated from each other (Fig. 1 ▸). These subunits are separated by isolated FeS_4_ tetra­hedra and surrounded by a snaking, three-dimensional network of corner-sharing CuS_4_ tetra­hedra that serve to link the (Ge_2_S_7_)^6−^ and FeS_4_ subunits. All of the tetra­hedra are aligned along one crystallographic direction, rendering the structure non-centrosymmetric (Fig. 2 ▸). All DLSs exhibit a honeycomb pattern in their crystal structure (Fig. 3 ▸); the various resulting space groups arise from the different possible cation-ordering patterns.

Selected geometrical data for (I) and (II) are given in Tables 1 ▸ and 2 ▸, respectively. The average Fe—S (I) and Co—S (II) bond distances are 2.334 (6) and 2.317 (6) Å, respectively. These values align well with other compounds containing iron or cobalt tetra­hedrally coordinated by sulfur. For example, the average Fe—S distance found in Li_2_FeGeS_4_ is 2.34 (2) Å (Brant, dela Cruz *et al.*, 2014 ▸), while the average Co—S distance found in Li_2_CoGeS_4_ is 2.31 (3) Å (Brant, Devlin *et al.*, 2015 ▸). The average Ge—S distances are 2.240 (4) and 2.244 (5) Å for (I) and (II), respectively. These distances are also close to those of the lithium-containing DLSs: Li_2_FeGeS_4_ (Brant, Devlin *et al.*, 2015 ▸) and Li_2_CoGeS_4_ (Brant, dela Cruz *et al.*, 2014 ▸) possess values of 2.23 (2) and 2.22 (3) Å, respectively. The average tetra­hedral bond angles for all cations in both title compounds is, within uncertainty, ideal. For comparison, the tetra­hedral angular ranges encountered in Cu_2_FeGeS_4_ (Wintenberger, 1979 ▸) and Cu_2_CoGeS_4_ (Gulay *et al.*, 2004 ▸) are 109.471–109.484° and 109.473–109.579°, respectively. The sulfur anions also exhibit tetra­hedral coordination. Both S2 and S4 are coordinated by two copper, one germanium and one iron or cobalt cation. S1 is connected to two germanium and two copper cations, while S3 is surrounded by one germanium and three copper cations.

## Database survey   

Quaternary DLSs exist with several different formulae; examples that incorporate chalcogenides as the anion are I–II_2_–III–VI_4_, I_2_–II–IV–VI_4_, and I_4_–II–IV_2_–VI_7_. In these formulae, the Roman numerals represent the number of valence electrons for each element. Compounds of the formula I–II_2_–III–VI_4_, such as CuMn_2_InS_4_ (Delgado & Sagredo, 2016 ▸) and CuFe_2_InSe_4_ (Delgado *et al.*, 2008 ▸) include trivalent elements, while the other relevant formulae mentioned above, including the title compounds, contain tetra­valent elements. Numerous DLSs of the general formula I_2_–II–IV–VI_4_ have been reported and crystallize in non-centrosymmetric space groups, such as *I* 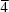
2*m* and *Pmn*2_1_ with the stannite and wurtz-stannite structure types that are derived from the cubic and hexa­gonal diamond structures, respectively (Brunetta *et al.*, 2013 ▸). The monovalent ions incorporated in these materials include Li (Wu, Zhang, *et al.*, 2017 ▸; Wu & Pan, 2017 ▸), Cu (Parthé *et al.*, 1969 ▸) or Ag (Brunetta *et al.*, 2013 ▸) and the divalent ions include a number of metals, such as Mg (Liu *et al.*, 2013 ▸), Mn (Bernert & Pfitzner, 2005 ▸), Fe (Wintenberger, 1979 ▸), Co (Bernert and Pfitzner 2006 ▸), Zn (Parasyuk *et al.*, 2001 ▸), Cd (Rosmus *et al.*, 2014 ▸) and Hg (Olekseyuk *et al.*, 2005 ▸). The tetra­valent ions found in these compounds are usually Si, Ge, or Sn, while the hexa­valent atoms (*i.e.*, the divalent anions) can be S (Lekse *et al.*, 2009 ▸), Se (Gulay, Romanyuk & Parasyuk, 2002 ▸), or Te (Parasyuk *et al.*, 2005 ▸). Some specific examples include Cu_2_MgGeS_4_ (Liu *et al.*, 2013 ▸) and Ag_2_MnSnS_4_ (Friedrich *et al.*, 2018 ▸).

In contrast, considerably fewer compounds of the general formula I_4_–II–IV_2_–VI_7_ have been discovered: only seven of these, which crystallize in either space group *C*2 or *Cc* with structures derived from cubic or hexa­gonal diamond, respectively, have been published to date: Li_4_MnGe_2_S_7_ (*Cc*) (Kaib *et al.*, 2013 ▸), Li_4_MnSn_2_Se_7_ (*Cc*) (Kaib *et al.*, 2013 ▸), Li_4_HgGe_2_S_7_ (*Cc*) (Wu, Yang, Pan 2017 ▸), Ag_4_HgGe_2_S_7_ (*Cc*) (Gulay, Olekseyuk & Parasyuk 2002 ▸), Ag_4_CdGe_2_S_7_ (*Cc*) (Gulay, Olekseyuk & Parasyuk 2002 ▸), Cu_4_NiSi_2_S_7_ (*C*2) (Schäfer *et al.*, 1980 ▸), and Cu_4_NiGe_2_S_7_ (*C*2) (Schäfer *et al.*, 1980 ▸).

## X-ray powder diffraction and thermal analysis   

**Figure 4:**
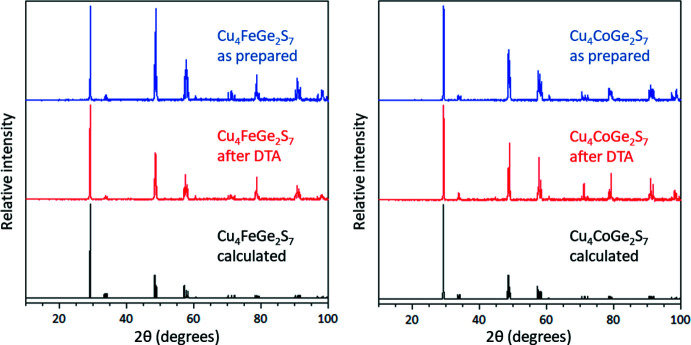
Comparison of experimental X-ray powder diffraction patterns before and after DTA with those calculated using the single-crystal structures for Cu_4_FeGe_2_S_7_ (left) and Cu_4_CoGe_2_S_7_ (right).

The calculated and observed X-ray powder diffraction patterns match well (Fig. 4 ▸), indicating that the title compounds are the major phases of the respective reactions. An optimization of the synthetic protocol is needed to isolate the desired phases in phase-pure form.

**Figure 5:**
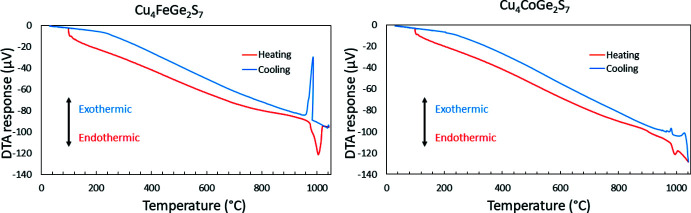
Differential thermal analysis diagrams obtained for the title compounds showing the melting and recrystallization events.

Differential thermal analysis (DTA) reveals that Cu_4_FeGe_2_S_7_ and Cu_4_CoGe_2_S_7_ show relatively high thermal stability and melting and recrystallization events with appropriate hysteresis around 1000°C (Fig. 5 ▸). Multiple heating-cooling cycles for each sample were consistent, suggesting that the thermal events are reversible. X-ray powder diffraction of the DTA residues indicated that the samples were not changed by the thermal analyses, implying that they melt congruently. DTA also suggests that neither compound is a single phase, as there are some small shoulders on the peaks indicative of the thermal events.

## Materials and methods   

All powdered elements were acquired from commercial suppliers and used as obtained with the exception of germanium metal, which was purchased as chunks and ground to a fine powder using a Diamonite™ mortar and pestle prior to use. Powder X-ray diffraction data were recorded from 10–100° 2θ using a PANalytical X’Pert Pro MPD powder X-ray diffractometer operating with Cu *K*_α_ radiation (λ = 1.541871 Å), a tube power of 45 kV and 40 mA and a step size of 0.017°. DTA data were obtained using a Shimadzu DTA50 thermal analyzer. Each sample was vacuum-sealed in a fused-silica ampoule, placed alongside an ampoule containing an Al_2_O_3_ reference of comparable mass, heated from room temperature to 1050°C at a rate of 10°C min^−1^ and subsequently cooled to room temperature at the same rate. A second heating–cooling cycle was conducted in order to determine the reproducibility of the thermal events.

## Synthesis and crystallization   

Cu_4_FeGe_2_S_7_ and Cu_4_CoGe_2_S_7_ were synthesized by combining stoichiometric amounts of Cu (99.999%), Fe (99.99%) or Co (99.99%), Ge (99.999%) and S (99.5%, sublimed) powders. The powders were mixed and placed into 12 mm o.d. fused-silica tubes that were subsequently attached to a vacuum line, evacuated and flame sealed. The reaction vessels were placed upright into ceramic containers inside programmable furnaces, where they were heated to 1000°C in 24 h, held there for 48 h, cooled to 900°C over the course of 50 h, and held there for 96 h, before being allowed to cool to room temperature over a 24 h period. Subsequently, the reaction vessels were cut open and the contents were examined under a light microscope. The products consisted of loose silvery gray microcrystalline powders from which small single crystals were selected for single-crystal X-ray diffraction.

## Refinement   

Crystal data, data collection parameters, and structure refinement details are summarized in Table 3 ▸. Extinction parameters were refined for each compound. After the final refinement, the Flack parameter for both structures refined to 0.06 (3), indicating that the absolute structure is correct. In Cu_4_FeGe_2_S_7_, the largest difference peak is located 1.15 Å from Cu2 while the deepest difference hole is 1.50 Å from S3. For Cu_4_CoGe_2_S_7_, the largest difference peak is 0.67 Å from Co while the deepest difference hole is 0.83 Å from Ge.

## Supplementary Material

Crystal structure: contains datablock(s) I, II, global. DOI: 10.1107/S2056989020007872/hb7920sup1.cif

Structure factors: contains datablock(s) I. DOI: 10.1107/S2056989020007872/hb7920Isup2.hkl

Structure factors: contains datablock(s) II. DOI: 10.1107/S2056989020007872/hb7920IIsup3.hkl

CCDC references: 2009145, 2009144

Additional supporting information:  crystallographic information; 3D view; checkCIF report

## Figures and Tables

**Table 1:** Selected bond lengths (Å) for (I)

Cu1—S3^i^	2.290 (2)	Fe—S4^v^	2.331 (3)
Cu1—S3	2.302 (2)	Fe—S4	2.331 (3)
Cu1—S2^ii^	2.303 (2)	Fe—S2^iv^	2.337 (3)
Cu1—S1^iii^	2.353 (2)	Fe—S2^vi^	2.337 (3)
Cu2—S3	2.294 (2)	Ge—S3^vii^	2.196 (2)
Cu2—S2	2.309 (2)	Ge—S2	2.221 (2)
Cu2—S4^iv^	2.309 (2)	Ge—S4^iii^	2.233 (2)
Cu2—S4	2.319 (2)	Ge—S1^iii^	2.3107 (19)

Symmetry codes: (i) 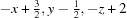; (ii) 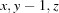; (iii) 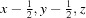; (iv) 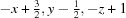; (v) 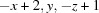; (vi) 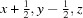; (vii) 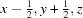.

**Table 2:** Selected bond lengths (Å) for (II)

Cu1—S3^i^	2.292 (2)	Co—S4^v^	2.308 (3)
Cu1—S2^ii^	2.295 (2)	Co—S4	2.308 (3)
Cu1—S3	2.304 (2)	Co—S2^iv^	2.325 (3)
Cu1—S1^iii^	2.343 (3)	Co—S2^vi^	2.325 (3)
Cu2—S3	2.290 (2)	Ge—S2	2.211 (3)
Cu2—S2	2.298 (3)	Ge—S3^vii^	2.211 (2)
Cu2—S4^iv^	2.301 (2)	Ge—S4^iii^	2.236 (2)
Cu2—S4	2.311 (2)	Ge—S1^iii^	2.316 (2)

Symmetry codes: (i) 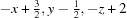; (ii) 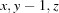; (iii) 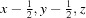; (iv) 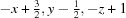; (v) 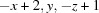; (vi) 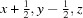; (vii) 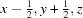.

**Table 3:** Experimental details

	(I)	(II)
Crystal data		
Chemical formula	Cu_4_FeGe_2_S_7_	Cu_4_CoGe_2_S_7_
*M*_r_	679.61	682.69
Crystal system, space group	Monoclinic, *C*2	Monoclinic, *C*2
Temperature (K)	296	296
*a*, *b*, *c* (Å)	11.7405 (6), 5.3589 (2), 8.3420 (4)	11.7280 (2), 5.33987 (10), 8.33133 (14)
β (°)	98.661 (3)	98.6680 (12)
*V* (Å^3^)	518.86 (4)	515.80 (2)
*Z*	2	2
Radiation type	Mo *K*α	Mo *K*α
μ (mm^−1^)	16.46	16.76
Crystal size (mm)	0.08 × 0.08 × 0.03	0.08 × 0.07 × 0.06

Data collection		
Diffractometer	Bruker SMART APEXII	Bruker SMART APEXII
Absorption correction	Multi-scan (*SADABS*; Sheldrick, 2002 ▸)	Multi-scan (*SADABS*; Sheldrick, 2002 ▸)
*T*_min_, *T*_max_	0.246, 0.435	0.356, 0.435
No. of measured, independent and observed [*I* > 2σ(*I*)] reflections	2258, 1187, 994	2239, 1177, 1039
*R*_int_	0.021	0.017
(sin θ/λ)_max_ (Å^−1^)	0.649	0.649

Refinement		
*R*[*F* ^2^ > 2σ(*F* ^2^)], *wR*(*F* ^2^), *S*	0.034, 0.094, 1.06	0.030, 0.075, 1.11
No. of reflections	1187	1177
No. of parameters	67	67
No. of restraints	1	1
Δρ_max_, Δρ_min_ (e Å^−3^)	0.75, −0.89	0.88, −0.49
Absolute structure	Flack (1983 ▸)	Flack (1983 ▸)
Absolute structure parameter	0.06 (3)	0.06 (3)

Computer programs: *SMART* and *SAINT* (Bruker, 1998 ▸), *SHELXS97* (Sheldrick, 2008 ▸), *SHELXL2018/3* (Sheldrick, 2015 ▸) and *CrystalMaker* (Palmer, 2014 ▸).

## supplementary crystallographic information

### Tetracopper iron digermanium heptasulfide (I) Crystal data

**Table:**

Cu_4_FeGe_2_S_7_	*F*(000) = 636
*M**_r_* = 679.61	*D*_x_ = 4.350 Mg m^−^^3^
Monoclinic, *C*2	Mo *K*α radiation, λ = 0.71073 Å
*a* = 11.7405 (6) Å	Cell parameters from 4891 reflections
*b* = 5.3589 (2) Å	θ = 4.2–31.4°
*c* = 8.3420 (4) Å	µ = 16.46 mm^−^^1^
β = 98.661 (3)°	*T* = 296 K
*V* = 518.86 (4) Å^3^	Irregular, grey
*Z* = 2	0.08 × 0.08 × 0.03 mm

### Tetracopper iron digermanium heptasulfide (I) Data collection

**Table:**

Bruker SMART APEXII diffractometer	994 reflections with *I* > 2σ(*I*)
φ and ω Scans scans	*R*_int_ = 0.021
Absorption correction: multi-scan (SADABS; Sheldrick, 2002)	θ_max_ = 27.5°, θ_min_ = 2.5°
*T*_min_ = 0.246, *T*_max_ = 0.435	*h* = −15→15
2258 measured reflections	*k* = −6→6
1187 independent reflections	*l* = −10→10

### Tetracopper iron digermanium heptasulfide (I) Refinement

**Table:**

Refinement on *F*^2^	*w* = 1/[σ^2^(*F*_o_^2^) + (0.0315*P*)^2^] where *P* = (*F*_o_^2^ + 2*F*_c_^2^)/3
Least-squares matrix: full	(Δ/σ)_max_ < 0.001
*R*[*F*^2^ > 2σ(*F*^2^)] = 0.034	Δρ_max_ = 0.75 e Å^−^^3^
*wR*(*F*^2^) = 0.094	Δρ_min_ = −0.89 e Å^−^^3^
*S* = 1.06	Extinction correction: SHELXL-2018/3 (Sheldrick 2018), Fc^*^=kFc[1+0.001xFc^2^λ^3^/sin(2θ)]^-1/4^
1187 reflections	Extinction coefficient: 0.0123 (9)
67 parameters	Absolute structure: Flack (1983)
1 restraint	Absolute structure parameter: 0.06 (3)
Primary atom site location: structure-invariant direct methods	

### Tetracopper iron digermanium heptasulfide (I) Special details

**Table:**

Geometry. All esds (except the esd in the dihedral angle between two l.s. planes) are estimated using the full covariance matrix. The cell esds are taken into account individually in the estimation of esds in distances, angles and torsion angles; correlations between esds in cell parameters are only used when they are defined by crystal symmetry. An approximate (isotropic) treatment of cell esds is used for estimating esds involving l.s. planes.
Refinement. Refined as a two-component inversion twin

### Tetracopper iron digermanium heptasulfide (I) Fractional atomic coordinates and isotropic or equivalent isotropic displacement parameters (Å^2^)

**Table:**

	*x*	*y*	*z*	*U*_iso_*/*U*_eq_	
Cu1	0.64298 (8)	0.2054 (3)	0.93484 (11)	0.0174 (7)	
Cu2	0.71147 (8)	0.7098 (2)	0.64229 (10)	0.0165 (6)	
Fe	1.000000	0.7200 (6)	0.500000	0.0112 (6)	
Ge	0.42524 (6)	0.7285 (3)	0.78371 (7)	0.0092 (3)	
S1	1.000000	0.9789 (6)	1.000000	0.0091 (6)	
S2	0.56834 (15)	0.9593 (5)	0.71791 (18)	0.0111 (6)	
S3	0.78390 (14)	0.4527 (4)	0.85310 (17)	0.0091 (6)	
S4	0.85688 (15)	0.9704 (5)	0.58180 (19)	0.0101 (5)	

### Tetracopper iron digermanium heptasulfide (I) Atomic displacement parameters (Å^2^)

**Table:**

	*U*^11^	*U*^22^	*U*^33^	*U*^12^	*U*^13^	*U*^23^
Cu1	0.0171 (6)	0.0169 (15)	0.0184 (6)	−0.0019 (5)	0.0032 (4)	0.0025 (5)
Cu2	0.0171 (6)	0.0142 (13)	0.0185 (6)	0.0005 (8)	0.0037 (4)	0.0017 (4)
Fe	0.0115 (8)	0.0137 (17)	0.0093 (7)	0.000	0.0041 (6)	0.000
Ge	0.0080 (5)	0.0100 (8)	0.0096 (5)	−0.0009 (8)	0.0018 (3)	−0.0007 (4)
S1	0.0095 (13)	0.0093 (16)	0.0087 (11)	0.000	0.0014 (9)	0.000
S2	0.0089 (11)	0.0128 (15)	0.0120 (9)	−0.0029 (10)	0.0030 (7)	0.0009 (9)
S3	0.0081 (11)	0.0076 (14)	0.0122 (9)	0.0004 (8)	0.0033 (8)	−0.0003 (8)
S4	0.0101 (10)	0.0094 (13)	0.0107 (9)	−0.0012 (7)	0.0010 (7)	−0.0004 (10)

### Tetracopper iron digermanium heptasulfide (I) Geometric parameters (Å, º)

**Table:**

Cu1—S3^i^	2.290 (2)	Fe—S4^v^	2.331 (3)
Cu1—S3	2.302 (2)	Fe—S4	2.331 (3)
Cu1—S2^ii^	2.303 (2)	Fe—S2^iv^	2.337 (3)
Cu1—S1^iii^	2.353 (2)	Fe—S2^vi^	2.337 (3)
Cu2—S3	2.294 (2)	Ge—S3^vii^	2.196 (2)
Cu2—S2	2.309 (2)	Ge—S2	2.221 (2)
Cu2—S4^iv^	2.309 (2)	Ge—S4^iii^	2.233 (2)
Cu2—S4	2.319 (2)	Ge—S1^iii^	2.3107 (19)
			
S3^i^—Cu1—S3	111.52 (5)	Ge^viii^—S1—Ge^ix^	109.23 (13)
S3^i^—Cu1—S2^ii^	108.81 (12)	Ge^viii^—S1—Cu1^viii^	112.37 (4)
S3—Cu1—S2^ii^	107.60 (6)	Ge^ix^—S1—Cu1^viii^	109.92 (4)
S3^i^—Cu1—S1^iii^	112.79 (6)	Ge^viii^—S1—Cu1^ix^	109.92 (4)
S3—Cu1—S1^iii^	106.23 (13)	Ge^ix^—S1—Cu1^ix^	112.37 (4)
S2^ii^—Cu1—S1^iii^	109.75 (6)	Cu1^viii^—S1—Cu1^ix^	102.96 (15)
S3—Cu2—S2	109.84 (6)	Ge—S2—Cu1^x^	109.71 (8)
S3—Cu2—S4^iv^	109.30 (11)	Ge—S2—Cu2	110.72 (12)
S2—Cu2—S4^iv^	111.38 (6)	Cu1^x^—S2—Cu2	109.87 (7)
S3—Cu2—S4	109.17 (7)	Ge—S2—Fe^vii^	109.96 (9)
S2—Cu2—S4	107.49 (11)	Cu1^x^—S2—Fe^vii^	108.33 (14)
S4^iv^—Cu2—S4	109.62 (5)	Cu2—S2—Fe^vii^	108.21 (7)
S4^v^—Fe—S4	109.72 (19)	Ge^vi^—S3—Cu1^viii^	108.48 (7)
S4^v^—Fe—S2^iv^	107.13 (6)	Ge^vi^—S3—Cu2	109.52 (7)
S4—Fe—S2^iv^	113.18 (6)	Cu1^viii^—S3—Cu2	106.84 (11)
S4^v^—Fe—S2^vi^	113.18 (6)	Ge^vi^—S3—Cu1	111.62 (11)
S4—Fe—S2^vi^	107.13 (6)	Cu1^viii^—S3—Cu1	108.31 (7)
S2^iv^—Fe—S2^vi^	106.58 (17)	Cu2—S3—Cu1	111.89 (8)
S3^vii^—Ge—S2	112.97 (13)	Ge^ix^—S4—Cu2^xi^	107.94 (11)
S3^vii^—Ge—S4^iii^	109.74 (6)	Ge^ix^—S4—Cu2	113.68 (8)
S2—Ge—S4^iii^	110.97 (6)	Cu2^xi^—S4—Cu2	109.46 (7)
S3^vii^—Ge—S1^iii^	108.92 (5)	Ge^ix^—S4—Fe	112.60 (9)
S2—Ge—S1^iii^	107.64 (6)	Cu2^xi^—S4—Fe	105.12 (7)
S4^iii^—Ge—S1^iii^	106.34 (12)	Cu2—S4—Fe	107.68 (14)

Symmetry codes: (i) −*x*+3/2, *y*−1/2, −*z*+2; (ii) *x*, *y*−1, *z*; (iii) *x*−1/2, *y*−1/2, *z*; (iv) −*x*+3/2, *y*−1/2, −*z*+1; (v) −*x*+2, *y*, −*z*+1; (vi) *x*+1/2, *y*−1/2, *z*; (vii) *x*−1/2, *y*+1/2, *z*; (viii) −*x*+3/2, *y*+1/2, −*z*+2; (ix) *x*+1/2, *y*+1/2, *z*; (x) *x*, *y*+1, *z*; (xi) −*x*+3/2, *y*+1/2, −*z*+1.

### Tetracopper cobalt digermanium heptasulfide (II) Crystal data

**Table:**

Cu_4_CoGe_2_S_7_	*F*(000) = 638
*M**_r_* = 682.69	*D*_x_ = 4.396 Mg m^−^^3^
Monoclinic, *C*2	Mo *K*α radiation, λ = 0.71073 Å
*a* = 11.7280 (2) Å	Cell parameters from 4827 reflections
*b* = 5.33987 (10) Å	θ = 4.2–32.6°
*c* = 8.33133 (14) Å	µ = 16.76 mm^−^^1^
β = 98.6680 (12)°	*T* = 296 K
*V* = 515.80 (2) Å^3^	Irregular, grey
*Z* = 2	0.08 × 0.07 × 0.06 mm

### Tetracopper cobalt digermanium heptasulfide (II) Data collection

**Table:**

Bruker SMART APEXII diffractometer	1039 reflections with *I* > 2σ(*I*)
φ and ω Scans scans	*R*_int_ = 0.017
Absorption correction: multi-scan (SADABS; Sheldrick, 2002)	θ_max_ = 27.5°, θ_min_ = 2.5°
*T*_min_ = 0.356, *T*_max_ = 0.435	*h* = −15→15
2239 measured reflections	*k* = −6→6
1177 independent reflections	*l* = −10→10

### Tetracopper cobalt digermanium heptasulfide (II) Refinement

**Table:**

Refinement on *F*^2^	*w* = 1/[σ^2^(*F*_o_^2^) + 3.9921*P*] where *P* = (*F*_o_^2^ + 2*F*_c_^2^)/3
Least-squares matrix: full	(Δ/σ)_max_ < 0.001
*R*[*F*^2^ > 2σ(*F*^2^)] = 0.030	Δρ_max_ = 0.88 e Å^−^^3^
*wR*(*F*^2^) = 0.075	Δρ_min_ = −0.49 e Å^−^^3^
*S* = 1.11	Extinction correction: *SHELXL2018/3* (Sheldrick 2015), Fc^*^=kFc[1+0.001xFc^2^λ^3^/sin(2θ)]^-1/4^
1177 reflections	Extinction coefficient: 0.060 (3)
67 parameters	Absolute structure: Flack (1983)
1 restraint	Absolute structure parameter: 0.06 (3)
Primary atom site location: structure-invariant direct methods	

### Tetracopper cobalt digermanium heptasulfide (II) Special details

**Table:**

Geometry. All esds (except the esd in the dihedral angle between two l.s. planes) are estimated using the full covariance matrix. The cell esds are taken into account individually in the estimation of esds in distances, angles and torsion angles; correlations between esds in cell parameters are only used when they are defined by crystal symmetry. An approximate (isotropic) treatment of cell esds is used for estimating esds involving l.s. planes.
Refinement. Refined as a two-component inversion twin

### Tetracopper cobalt digermanium heptasulfide (II) Fractional atomic coordinates and isotropic or equivalent isotropic displacement parameters (Å^2^)

**Table:**

	*x*	*y*	*z*	*U*_iso_*/*U*_eq_	
Cu1	0.64303 (9)	0.2051 (4)	0.93412 (12)	0.0177 (7)	
Cu2	0.71154 (9)	0.7108 (2)	0.64143 (12)	0.0158 (6)	
Co	1.000000	0.7206 (7)	0.500000	0.0141 (8)	
Ge	0.42703 (6)	0.7276 (4)	0.78209 (9)	0.0092 (4)	
S1	1.000000	0.9762 (6)	1.000000	0.0090 (6)	
S2	0.56898 (15)	0.9604 (5)	0.7167 (2)	0.0118 (6)	
S3	0.78425 (15)	0.4533 (4)	0.8520 (2)	0.0092 (6)	
S4	0.85742 (15)	0.9682 (5)	0.5803 (2)	0.0094 (5)	

### Tetracopper cobalt digermanium heptasulfide (II) Atomic displacement parameters (Å^2^)

**Table:**

	*U*^11^	*U*^22^	*U*^33^	*U*^12^	*U*^13^	*U*^23^
Cu1	0.0181 (6)	0.0182 (18)	0.0167 (6)	−0.0026 (5)	0.0023 (4)	0.0018 (6)
Cu2	0.0168 (6)	0.0131 (14)	0.0175 (6)	0.0004 (8)	0.0029 (4)	0.0014 (6)
Co	0.0118 (7)	0.021 (2)	0.0096 (7)	0.000	0.0028 (5)	0.000
Ge	0.0090 (5)	0.0100 (9)	0.0086 (5)	−0.0011 (7)	0.0012 (3)	−0.0019 (6)
S1	0.0102 (12)	0.0092 (18)	0.0072 (11)	0.000	−0.0001 (9)	0.000
S2	0.0101 (9)	0.0160 (15)	0.0097 (9)	−0.0022 (11)	0.0028 (7)	−0.0007 (9)
S3	0.0095 (9)	0.0076 (16)	0.0109 (9)	0.0012 (8)	0.0025 (7)	0.0017 (9)
S4	0.0113 (9)	0.0090 (13)	0.0076 (8)	−0.0008 (7)	0.0008 (7)	−0.0012 (10)

### Tetracopper cobalt digermanium heptasulfide (II) Geometric parameters (Å, º)

**Table:**

Cu1—S3^i^	2.292 (2)	Co—S4^v^	2.308 (3)
Cu1—S2^ii^	2.295 (2)	Co—S4	2.308 (3)
Cu1—S3	2.304 (2)	Co—S2^iv^	2.325 (3)
Cu1—S1^iii^	2.343 (3)	Co—S2^vi^	2.325 (3)
Cu2—S3	2.290 (2)	Ge—S2	2.211 (3)
Cu2—S2	2.298 (3)	Ge—S3^vii^	2.211 (2)
Cu2—S4^iv^	2.301 (2)	Ge—S4^iii^	2.236 (2)
Cu2—S4	2.311 (2)	Ge—S1^iii^	2.316 (2)
			
S3^i^—Cu1—S2^ii^	109.37 (12)	Ge^viii^—S1—Ge^ix^	109.14 (15)
S3^i^—Cu1—S3	111.62 (6)	Ge^viii^—S1—Cu1^viii^	111.56 (4)
S2^ii^—Cu1—S3	107.25 (7)	Ge^ix^—S1—Cu1^viii^	110.41 (4)
S3^i^—Cu1—S1^iii^	112.08 (6)	Ge^viii^—S1—Cu1^ix^	110.41 (4)
S2^ii^—Cu1—S1^iii^	109.73 (7)	Ge^ix^—S1—Cu1^ix^	111.56 (4)
S3—Cu1—S1^iii^	106.65 (13)	Cu1^viii^—S1—Cu1^ix^	103.68 (17)
S3—Cu2—S2	109.96 (7)	Ge—S2—Cu1^x^	109.59 (8)
S3—Cu2—S4^iv^	108.79 (11)	Ge—S2—Cu2	110.30 (13)
S2—Cu2—S4^iv^	111.31 (7)	Cu1^x^—S2—Cu2	109.95 (8)
S3—Cu2—S4	108.88 (8)	Ge—S2—Co^vii^	109.91 (9)
S2—Cu2—S4	107.97 (11)	Cu1^x^—S2—Co^vii^	108.57 (14)
S4^iv^—Cu2—S4	109.90 (5)	Cu2—S2—Co^vii^	108.48 (8)
S4^v^—Co—S4	110.11 (19)	Ge^vi^—S3—Cu2	109.57 (8)
S4^v^—Co—S2^iv^	107.45 (6)	Ge^vi^—S3—Cu1^viii^	108.46 (8)
S4—Co—S2^iv^	112.63 (7)	Cu2—S3—Cu1^viii^	107.17 (11)
S4^v^—Co—S2^vi^	112.63 (7)	Ge^vi^—S3—Cu1	111.81 (11)
S4—Co—S2^vi^	107.45 (6)	Cu2—S3—Cu1	111.81 (8)
S2^iv^—Co—S2^vi^	106.59 (18)	Cu1^viii^—S3—Cu1	107.84 (7)
S2—Ge—S3^vii^	112.75 (14)	Ge^ix^—S4—Cu2^xi^	107.42 (11)
S2—Ge—S4^iii^	111.49 (7)	Ge^ix^—S4—Co	111.98 (10)
S3^vii^—Ge—S4^iii^	109.30 (7)	Cu2^xi^—S4—Co	105.80 (7)
S2—Ge—S1^iii^	108.43 (7)	Ge^ix^—S4—Cu2	113.66 (9)
S3^vii^—Ge—S1^iii^	108.34 (6)	Cu2^xi^—S4—Cu2	109.24 (7)
S4^iii^—Ge—S1^iii^	106.29 (12)	Co—S4—Cu2	108.42 (14)

Symmetry codes: (i) −*x*+3/2, *y*−1/2, −*z*+2; (ii) *x*, *y*−1, *z*; (iii) *x*−1/2, *y*−1/2, *z*; (iv) −*x*+3/2, *y*−1/2, −*z*+1; (v) −*x*+2, *y*, −*z*+1; (vi) *x*+1/2, *y*−1/2, *z*; (vii) *x*−1/2, *y*+1/2, *z*; (viii) −*x*+3/2, *y*+1/2, −*z*+2; (ix) *x*+1/2, *y*+1/2, *z*; (x) *x*, *y*+1, *z*; (xi) −*x*+3/2, *y*+1/2, −*z*+1.
